# Prospective Audit and Feedback by Antibiotic Stewardship Teams to Reduce Antibiotic Overuse at Hospital Discharge: A Stepped-Wedge Cluster-Randomized Clinical Trial

**DOI:** 10.1001/jamanetworkopen.2025.49655

**Published:** 2026-01-09

**Authors:** Daniel J. Livorsi, Alyssa M. Thompson, Melissa S. Green, Angela C. Hoelscher, Kailye K. Chu, Elizabeth Neuner, Yvonne Burnett, Teri Hopkins, Elizabeth Walter, Rohini Dave, Ravi Tripathi, Haylie Lohmar, Andrew Dysangco, Kelly Percival, Dilek Ince, Jessica Kolkmeyer, Helen Newland, Michael Joshua Hendrix, Gosia Clore, Cody Poe, Amy O’Shea, Joseph Tholany, Kunatum Prasidthrathsint, Erin Rachmiel, Jahnavi Bongu, Alice Bewley, Kevin Hsueh

**Affiliations:** 1 Department of Medicine, University of Iowa Carver College of Medicine, Iowa City; 2 Center for Access and Delivery Research and Evaluation, Iowa City Veterans Affairs Health Care System, Iowa City, Iowa; 3 Barnes-Jewish West County Hospital, Creve Coeur, Missouri; 4 Progress West Hospital, O’Fallon, Missouri; 5 Barnes-Jewish Hospital, St Louis, Missouri; 6 Missouri Baptist Medical Center, St Louis, Missouri; 7 Audie L. Murphy Memorial Veterans’ Hospital, San Antonio, Texas; 8 Veterans Administration Maryland Health Care System, Baltimore, Maryland; 9 Richard L. Roudebush Veterans Administration Medical Center, Indianapolis, Indiana; 10 Department of Pharmaceutical Care, University of Iowa Health Care, Iowa City; 11 Christian Hospital, St Louis, Missouri; 12 BJC HealthCare, St Louis, Missouri; 13 Barnes Jewish St Peter’s Hospital, St Peters, Missouri; 14 Department of Medicine, Division of Infectious Diseases, Washington University School of Medicine, St Louis, Missouri; 15 University of Iowa Health Care, Iowa City

## Abstract

**Importance:**

Antibiotics prescribed at hospital discharge are frequently unnecessary or suboptimal. Strategies to improve prescribing are not well defined.

**Objective:**

To evaluate whether a discharge-focused prospective audit and feedback process decreases antibiotic overuse at hospital discharge.

**Design, Setting, and Participants:**

This stepped-wedge cluster-randomized clinical trial was conducted across participating units at 10 hospitals with antibiotic stewardship (AS) teams and supporting staff from December 5, 2022, to November 17, 2023. After a 24-week baseline period, 1 hospital crossed into the intervention arm every 2 weeks.

**Intervention:**

The intervention consisted of disseminating institutional guidelines for oral antibiotic step-down to frontline prescribers and conducting a prospective audit and feedback process for inpatients receiving antibiotics with an anticipated discharge date in the next 48 hours.

**Main Outcomes and Measures:**

The primary outcome was postdischarge antibiotic use. Secondary outcomes included inpatient antibiotic use, length of hospital stay, and readmission. Manual electronic health record reviews were performed in 434 cases to assess optimal antibiotic prescribing at discharge for patients who met specific criteria. Analysis was performed on a per-protocol basis.

**Results:**

There were 21 842 patient admissions (baseline, 14 288; intervention, 7554) across 10 hospitals. The median (IQR) age was 66 (53-75) years, with 13 380 (61.3%) males. At the hospital level, the mean (SD) number of patients audited by the AS team per week was 19.9 (5.8); approximately one-quarter of these audits (mean [SD], 5.0 [2.6]) resulted in feedback to the frontline prescribers. There were 3133 patients (21.9%) prescribed postdischarge antibiotics at baseline compared with 1645 patients (21.8%) during the intervention (odds ratio, 0.94 [95% CI, 0.84-1.05]). The mean (SD) postdischarge antibiotic duration was 7.1 (5.2) days at baseline compared with 7.6 (5.6) days during the intervention (mean difference, 0.02 [95% CI, −0.50 to 0.53] days). There were no statistical differences during the intervention compared with baseline for inpatient antibiotic duration (mean [SD], 4.4 (3.6) vs 4.2 [3.5] days; mean difference, 0.04 [95% CI, −0.20 to 0.27] days), length of hospital stay (mean [SD], 5.4 [4.8] vs 5.4 [5.0] days; mean difference 0.11 [95% CI, −0.12 to 0.33] days), or hospital readmission within 30 days (odds ratio, 1.02 [95% CI, 0.88-1.18]). Optimal antibiotic prescribing was more common during the intervention (122 of 264 cases [46.2%] vs 100 of 170 cases [58.8%]; odds ratio, 1.61 [95% CI, 1.08-2.40]). A total of 112 inpatient frontline prescribers were sent a postintervention survey; 40 (35.7%) responded, and 34 of 36 (94.4%) believed that the initiative improved antibiotic prescribing at hospital discharge.

**Conclusions and Relevance:**

In this stepped-wedged cluster-randomized clinical trial conducted across 10 hospitals, discharge-focused prospective audit and feedback did not decrease antibiotic use at hospital discharge but did improve optimal antibiotic prescribing for common and uncomplicated diagnoses. Other AS strategies are needed to decrease unnecessary antibiotic prescribing at this transition of care.

**Trial Registration:**

ClincialTrials.gov Identifier: NCT05471726

## Key Points

**Question:**

Does a discharge-focused prospective audit and feedback process decrease antibiotic overuse at hospital discharge?

**Findings:**

In this stepped-wedge cluster-randomized clinical trial across participating units at 10 hospitals with 21 842 admissions, the frequency and duration of antibiotic prescribing at hospital discharge did not decrease after implementing a prospective audit and feedback process. However, in selected patients with uncomplicated infections, optimal antibiotic-prescribing increased once the intervention went into effect.

**Meaning:**

Discharge-focused prospective audit and feedback was not effective in reducing general antibiotic overuse at hospital discharge, but it did improve antibiotic prescribing in a subset of patients, suggesting that other strategies are needed to prevent unnecessary antibiotic use at this transition of care.

## Teaser

This stepped-wedge cluster-randomized clinical trial investigates whether implementation of a discharge-focused prospective audit and feedback process decreases antibiotic overuse at hospital discharge.

## Introduction

Antibiotic stewardship (AS) programs often focus on measuring and improving inpatient antibiotic use, but at least 40% of all antibiotic exposure associated with an acute-care hospital stay is prescribed at the time of hospital discharge.^1,2,3^ Postdischarge antibiotics mediate clinical outcomes after discharge and may facilitate the spread of antibiotic resistance.

Postdischarge antibiotics are frequently unnecessary or suboptimal.^4,5,6,7^ Several nonrandomized trials have shown that auditing and providing feedback at discharge can improve antibiotic-prescribing at this transition of care.^8,9,10,11,12,13^

Herein we report a stepped-wedge cluster-randomized clinical trial to evaluate whether a discharge-focused approach to prospective audit and feedback can safely decrease antibiotic overuse at hospital discharge. A stepped-wedge design permitted rollout of this intervention across a large number of hospitals, which allowed an assessment of the implementation of the intervention in a variety of settings.

## Methods

The trial results are reported based on the Consolidated Standards of Reporting Trials (CONSORT) reporting guideline and the CONSORT Extension for stepped-wedge cluster-randomized clinical trials.^14^ This trial was registered on July, 13, 2022, with ClinicalTrials.gov, and the study protocol is provided in Supplement 1. A timeline of all study-related activities is shown in eTable 1 in Supplement 2. The institutional review boards of the University of Iowa and Washington University School of Medicine approved this study and granted a waiver of informed consent because the study was deemed to be minimal risk.

### Trial Design

We performed a stepped-wedge cluster-randomized clinical trial across 10 acute-care hospitals to evaluate the implementation and effectiveness of an AS intervention focused on hospital discharge. Each hospital (cluster) began the study (start date, December 5, 2022) in the control condition for a 24-week baseline period; once the baseline period ended, every 2 weeks (beginning May 22, 2023) 1 hospital sequentially crossed to the intervention arm. The order of crossover was randomly determined. By the end of the trial, all 10 hospitals were conducting the intervention (Figure). The trial ended on November 17, 2023. Our evaluation of this trial used data collected at 11 fixed time points (baseline and each of the 2-week long intervention periods).

**Figure.**
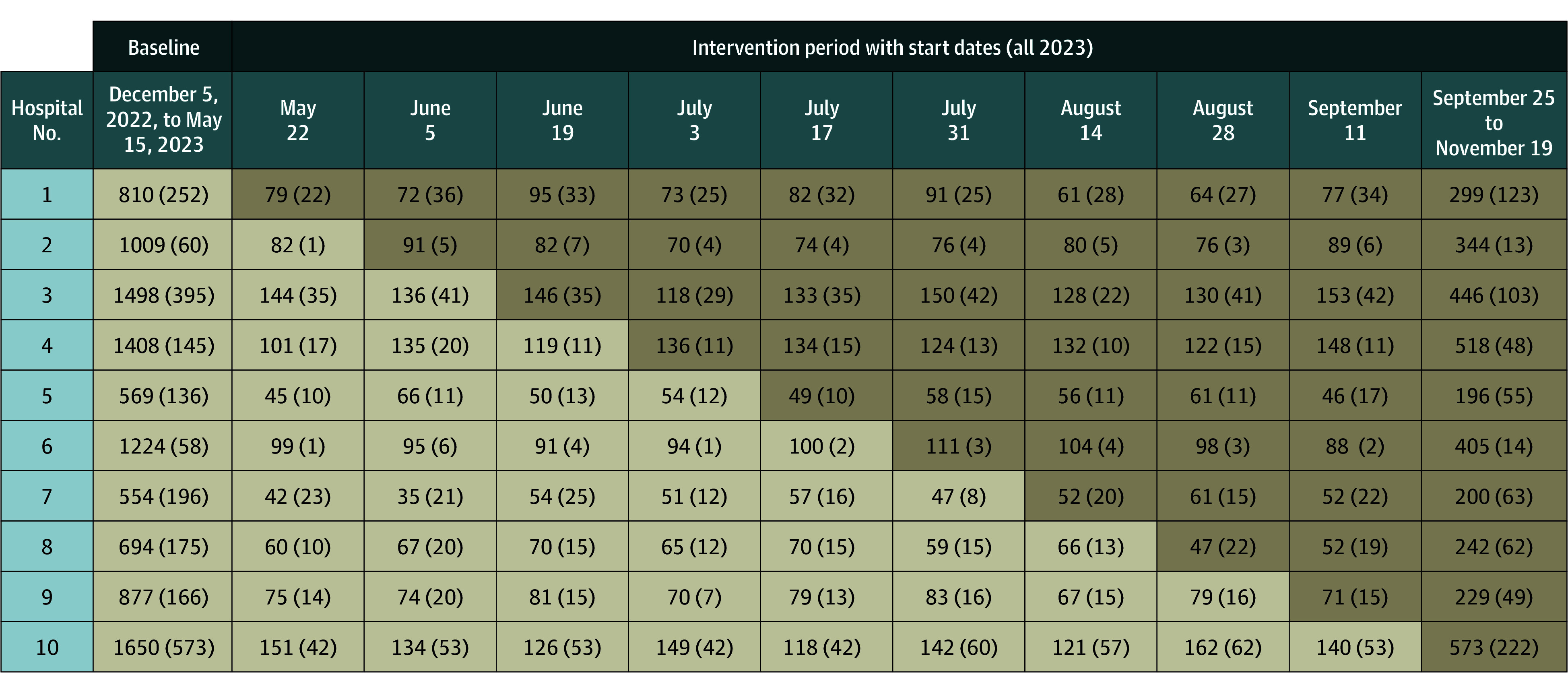
Diagram for the Stepped-Wedge Cluster-Randomized Clinical Trial For each of the 10 hospitals randomized, a tan box represents time spent in the baseline period; brown, time spent conducting the intervention. Each box shows the number of patient admissions during the time frames, and in parentheses, the number of patient admissions that were excluded.

### Participants

AS teams at acute-care hospitals were eligible to participate if they had not already implemented a prospective audit and feedback process focused on hospital discharge. Teams were recruited through national conference calls of the Prevention EpiCenter Program of the Centers for Disease Control and Prevention and known professional contacts of the lead author (D.J.L.).

Each AS team, which typically included both a physician and pharmacist champion, identified a group of inpatient units at their hospital to focus on with the intervention. Teams were asked to choose units that would, in aggregate, average at least 200 discharges per month. While all patients on these units were eligible for the intervention, outcomes were only measured for patients discharged to the community, as these patients would have electronically available data on postdischarge antibiotic use (see Outcomes). Patients who died or were transferred to another health care facility at discharge were excluded from the primary analysis.

### Interventions

The design of the intervention, which included 3 components, was decided on through consensus-building conversations with all sites. First, each AS team either developed new or updated existing institutional guidelines for oral antibiotic step-down therapy to treat common infections. Common infections were defined as acute exacerbations of chronic obstructive pulmonary disease, biliary tract or intra-abdominal infections with source control, pneumonia without abscess or empyema, skin and soft tissue infections, and urinary tract infections. Second, each AS team promoted the aforementioned guidelines and the goals of this project to participating units through face-to-face meetings with frontline prescribers and other key partners during the 1 to 2 months prior to the intervention starting at their site. Third, each AS team performed prospective audit and feedback during the intervention period for patients on participating units who were actively receiving inpatient antibiotics and who had an anticipated discharge in the next 48 hours. The process for identifying pending discharges varied by hospital (eTable 2 in Supplement 2). Depending on the hospital, either clinical pharmacists or stewardship champions led this auditing process, which sought to identify opportunities to improve antibiotic prescribing at discharge (dose, route, frequency, and duration) based on the aforementioned local guidelines during weekday working hours. The approach to providing real-time feedback to prescribers also varied by site (eTable 2 in Supplement 2). Fidelity to the audit and feedback intervention was measured through weekly REDCap (Research Electronic Data Capture) surveys.

### Outcomes

The primary effectiveness outcome of this study was postdischarge antibiotic use, which was quantified as the frequency at which postdischarge antibiotics were prescribed, and if prescribed, the postdischarge length of therapy, defined as the number of unique calendar days that the patient was prescribed an antibiotic to take after discharge. If patients were prescribed postdischarge antibiotics for 30 or more days, this duration did not contribute to the primary outcome, as such long durations likely reflected chronic suppression or prophylaxis. Secondary outcomes included inpatient length of antibiotic therapy, inpatient length of stay, 30-day hospital readmission rates, the incidence of *Clostridioides difficile* infections that occurred within 30 days after discharge, and the percentage of patients who were discharged on fluoroquinolones.

Manual electronic health record (EHR) reviews were performed for a subset of patients from each site to compare optimal antibiotic prescribing at discharge during the baseline period vs the intervention period. Patient admissions were eligible for the manual EHR review if they had a discharge diagnosis for 1 of the aforementioned common infections. Full inclusion and exclusion criteria are given in the eAppendix in Supplement 2. Optimal antibiotic prescribing was defined by both optimal selection and optimal duration of postdischarge antibiotic therapy.^15^

The Reach, Effectiveness, Adoption, Implementation, and Maintenance framework (RE-AIM) was used to comprehensively assess other aspects of the intervention.

- Reach was measured by tracking, on a weekly basis, the proportion of unique attending physicians on participating units and services who were either directly or indirectly recipients of feedback from the audit and feedback process during the intervention phase.
- Effectiveness was measured through the aforementioned primary and secondary outcomes.
- Adoption was measured through introductory calls and a baseline electronic survey of each participating AS team. Specifically, this outcome encompasses the context of the participating units and the reasons why each team chose to participate.
- Implementation was measured in a number of ways. First, weekly REDCap surveys were used to measure fidelity to and cost (ie, time commitment) of the intervention. Second, a postintervention survey was sent to frontline prescribers to assess the acceptability and feasibility of the intervention.
- Maintenance was measured by asking each AS team whether they were still performing the discharge-focused audit and feedback process 6 months after the trial ended.

### Sample Size

We calculated power for the primary outcome based on the following 5 assumptions: (1) an effect size of at least 10%; (2) an estimated intracluster correlation of 0.75; (3) a baseline mean postdischarge length of therapy of 135 days per 100 admissions, based on our pretrial measurements; (3) 100 discharges per site every 2 weeks; (4) a cluster-level SD of 3.4, based on our pretrial measurements; and (5) a type I error rate of 5%. Given these assumptions and using the stepped-wedge study design described, we estimated more than 90% power to determine a statistical difference in the primary outcome. For the manual EHR reviews, the sample size (300 cases from the baseline period and 200 from the intervention period) was estimated to provide 80% power at a 95% confidence level to detect a difference of 12.5 percentage points (62.5% vs 50%) in optimal antibiotic prescribing.

### Randomization

Randomization was performed at the hospital level, with all participating units and services having access to the intervention once the hospital entered the intervention phase. A simple randomization scheme using a random number generator was used by one of us (A.O.) to determine the order of implementation. Sites were not blinded to the order of implementation.

### Data Sources

We used administrative data to measure antibiotic use and patient characteristics. For the 3 Veterans Administration (VA) hospitals, these data were collected from the Corporate Data Warehouse via the VA Informatics and Computing Infrastructure. For the 7 non-VA sites, all patient data were collected from the EHR (Epic Systems Corporation). Missing data elements occurred in only 2 admissions; both elements were descriptive data and not necessary for the analyses.

### Statistical Analysis

Analyses were performed on a per-protocol basis. Primary and secondary outcomes were evaluated via a generalized linear mixed model and included step and intervention indicators as fixed effects and a random intercept for cluster to account for facility-level factors. This model structure provides proper weighting when cluster sizes vary and accounts for repeated measurements over time. Dichotomous outcomes used the binomial distribution and logit link, whereas continuous outcomes were modeled via the normal distribution with identity link. Odds ratios (ORs), mean differences, and 95% CIs are reported, as appropriate. Total antibiotic durations for specific infections are also reported, with mean differences assessed by the 2-sample *t* test. A 2-sided value of *P* < .05 was considered statistically significant.

## Results

The characteristics of the 10 participating hospitals are shown in Table 1. The median (IQR) number of total adult acute-care beds at each hospital was 190 (76-536). All hospitals had an inpatient infectious disease consultation service. Eight hospitals (80.0%) had been performing some form of prospective audit and feedback for inpatient antibiotics for the participating units during the 6 months prior to the intervention. Guidelines for oral antibiotic step-down were in place at 7 hospitals (70.0%) during the baseline period. All stewardship teams agreed that their motivation for adopting the intervention was the opportunity to improve antibiotic prescribing at the point of hospital discharge, which none of them had previously measured or addressed (Adoption).

**Table 1.** Baseline Characteristics of 10 Participating Hospitals

Hospital factors, antibiotic stewardship resources and processes	Response, No. (%)
Hospital characteristics	
No. of adult beds in entire hospital, median (IQR)^a^	190 (76-386)
Teaching hospital	7 (70.0)
Inpatient ID consultation	10 (100)
ID physician fellowship program	5 (50.0)
ID pharmacy training program	2 (20.0)
Antibiotic stewardship resources	
No. of FTEs for stewardship physician per 100 beds, median (IQR)	0.2 (0.1-0.3)
No. of FTEs for stewardship pharmacists per 100 beds, median (IQR)	0.2 (0.2-0.3)
Antibiotic stewardship expertise	
Leadership of antibiotic stewardship program	
Co-led by both pharmacist and physician	8 (80.0)
Pharmacist alone	2 (20.0)
Characteristics of stewardship physician-leader	
Was physically on site at the hospital	8 (80.0)
Completed an ID fellowship	8 (80.0)
Characteristics of Stewardship Pharmacist-leader	
Was physically on site at the hospital	10 (100)
Completed a PGY2 ID residency or ID fellowship	5 (50.0)
Antibiotic stewardship tools and processes	
Local guidelines for oral antibiotic step down were available^b^	7 (70.0)
Clinical pharmacists were assigned during the past 6 mos to the unit(s) that were participating in the trial	7 (70.0)
Antibiotics that require prior authorization or are restricted to the ID service on the unit(s) that are participating in the trial^c^	
Ciprofloxacin and levofloxacin (oral)	1 (10.0)
Cefepime	1 (10.0)
Vancomycin, piperacillin-tazobactam, or ceftriaxone	0
PAF for inpatient antibiotic use has been practiced during the past 6 mos on the unit(s) that participated in the trial	8 (80.0)
Stewardship team members and extenders involved in PAF at baseline^d^	
Stewardship physician	2 (25.0)
Stewardship pharmacist	7 (88.0)
Clinical pharmacist assigned to the unit or team	3 (38.0)
Frequency at which PAF was performed at baseline^d^	
Monday through Friday	6 (75.0)
Only on certain weekdays	2 (25.0)
Method of delivering feedback to prescribers at baseline^d^	
In person	4 (50.0)
Telephone	4 (50.0)
Electronic methods (eg, text page, email, EHR message)	8 (100)

Abbreviations: EHR, electronic health record; FTEs, full-time equivalents; ID, infectious disease; PAF, prospective audit and feedback; PGY, postgraduate year.

^a^ Three hospitals were Veterans Affairs medical centers, 2 were large university academic medical centers, and the remaining 5 were community hospitals.

^b^ If sites did not have guidelines developed at baseline, they implemented them as part of this study’s intervention. All 7 sites had guidelines that covered pneumonia (community- and hospital-acquired), intra-abdominal infections, skin and soft tissue infections, and urinary tract infections.

^c^ Prior authorization encompasses requiring approval prior to the first antibiotic dose or on the next business day.

^d^ Only 8 sites conducted PAF at baseline; thus the denominator for the percentage is 8.

For the intervention, AS teams chose to focus on 1 to 4 units (median, 2) that primarily cared for medicine patients. Each stewardship team implemented the intervention at their designated time point and continued to perform the study activities for the duration of their assigned intervention period. At the hospital-level, the mean (SD) number of unique patients audited by the AS team per week was 19.9 (16.6), and the mean (SD) number of unique patients for which feedback was given to frontline prescribers was 5.0 (2.6) per week (eTable 3 in Supplement 2).

In total, 1578 recommendations were made through the audit and feedback process. Recommendations addressed antibiotic duration in 487 cases (30.9%) and antibiotic selections in 386 cases (24.5%), followed by antibiotic dose (265 [16.8%]), route of administration (ie, intravenous to oral switch) (233 [14.8]%) and antibiotic cessation (207 [13.1]%). AS teams perceived that of all recommendations, 1426 (90.4%) had been accepted.

### Patients

After excluding 191 patients who died in the hospital and 4497 who were transferred to another health care facility, there were 21 842 patient admissions included in the analyses (14 288 in the baseline period and 7554 in the intervention period). The median (IQR) age of these patients was 66 (53-75) years, and 8462 (38.7%) were female and 13 380 (61.3%) male. During the baseline and intervention periods, 13 928 (97.5%) and 7326 (97.0%) patients, respectively, were treated by an inpatient internal medicine service (Table 2). Eight of every 10 patients were discharged on a weekday.

**Table 2.** Patient Characteristics During Baseline and Intervention Periods Across Participating Units From 10 Hospitals

Characteristic	Patient admissions, No. (%)		
	Total (n = 21 842)	Baseline (n = 14 288)	Intervention (n = 7554)
Age, median (IQR), y	66 (53-75)	66 (54-76)	65 (52-75)
Sex			
Female	8462 (38.7)	5429 (38.1)	3023 (40.0)
Male	13 380 (61.3)	8849 (61.9)	4531 (60.0)
Intensive care unit admission	2147 (9.8)	1461 (10.2)	686 (9.1)
Primary service			
Medicine	21 254 (97.3)	13 928 (97.5)	7326 (97.0)
Surgery	438 (2.0)	266 (1.9)	172 (2.3)
Neurology	123 (0.6)	77 (0.5)	46 (0.6)
Other	26 (0.1)	17 (0.1)	9 (0.1)
Comorbidity			
Peripheral vascular disease	5172 (23.7)	3529 (24.7)	1643 (21.8)
Chronic obstructive pulmonary disease	8047 (36.8)	5383 (37.7)	2664 (35.3)
Diabetes	6801 (31.1)	4514 (31.6)	2287 (30.3)
Kidney failure	7183 (32.9)	4831 (33.8)	2352 (31.1)
Liver disease, severe	1158 (5.3)	790 (5.5)	368 (4.9)
Congestive heart failure	6648 (30.4)	4495 (31.5)	2153 (28.5)
Arrhythmia	9155 (41.9)	5992 (41.9)	3163 (41.9)
Metastatic cancer	2008 (9.2)	1305 (9.1)	703 (9.3)
Alcohol use disorder	3432 (15.7)	2330 (16.3)	1102 (14.6)
Drug abuse disorder	3233 (14.8)	2175 (15.2)	1058 (14.0)
Malignant neoplasm	4980 (22.8)	3162 (22.1)	1818 (24.1)
Infection			
Respiratory	2799 (12.8)	1987 (13.9)	812 (10.8)
Skin and soft tissue	1513 (6.9)	976 (6.8)	537 (7.1)
Urinary tract	1534 (7.0)	977 (6.8)	557 (7.4)
Intra-abdominal or biliary	749 (3.4)	455 (3.2)	394 (3.9)
Discharge day			
Weekday	17 440 (79.8)	11 356 (79.5)	6084 (80.5)
Weekend	4402 (20.2)	2932 (20.5)	1470 (19.5)

#### Effectiveness

There were 3133 patients (21.9%) prescribed postdischarge antibiotics during the baseline period compared with 1645 patients (21.8%) during the intervention period (OR, 0.94 [95% CI, 0.84-1.05]) (Table 3). Among patients with an outpatient antibiotic prescribed, the mean (SD) postdischarge length of therapy was 7.1 (5.2) days at baseline compared with 7.6 (5.6) days during the intervention period (mean difference, 0.02 [95% CI, −0.50 to 0.53] days).

**Table 3.** Secondary Outcomes Across Participating Units From 10 Hospitals During Baseline and Intervention Periods

Outcome	Baseline, No. (%) or mean (SD)	Intervention, No. (%) or mean (SD)	OR or mean difference (95% CI) intervention compared with baseline
Postdischarge antibiotic exposure, No. (%)	3133 (21.9)	1645 (21.8)	OR: 0.94 (0.84 to 1.05)
Inpatient antibiotic exposure, No. (%)	7051 (49.4)	3871 (51.2)	OR: 1.00 (0.91 to 1.10)
Readmission within 30 d, No. (%)	1786 (12.5)	969 (12.8)	OR: 1.02 (0.88 to 1.18)
Fluoroquinolone at hospital discharge, No. (%)	740 (5.2)	379 (5.0)	OR: 0.94 (0.76 to 1.16)
*Clostridioides difficile* test positive, No. (%)	43 (0.3)	25 (0.3)	OR: 1.75 (0.75 to 4.08)
Postdischarge antibiotic length of therapy, mean (SD), d^a^	7.1 (5.2)	7.6 (5.6)	Mean difference: 0.02 (−0.50 to 0.53)
Inpatient antibiotic length of therapy, mean (SD), d^b^	4.4 (3.6)	4.2 (3.5)	Mean difference: 0.04 (−0.20 to 0.27)
Length of stay, mean (SD), d	5.4 (4.8)	5.4 (5.0)	Mean difference: 0.11 (−0.12 to 0.33)

Abbreviation: OR, odds ratio.

^a^ Postdischarge antibiotic length of therapy was only calculated in 3133 and 1645 patients who received postdischarge antibiotics at baseline and during the intervention period, respectively. Among these patients, mean (SD) total antibiotic length of therapy (inpatient plus postdischarge) was 11.2 (6.4) days at baseline and 11.8 (6.7) days during the intervention.

^b^ Inpatient antibiotic length of therapy was calculated only for patients who received inpatient antibiotics.

There were 7051 patients (49.4%) exposed to inpatient antibiotics during the baseline period compared with 3871 patients (51.2%) during the intervention period (OR, 1.00 [95% CI, 0.91-1.10]). Among those with an inpatient antibiotic prescribed, the mean (SD) inpatient length of therapy was 4.4 (3.6) days at baseline compared with 4.2 (3.5) days during the intervention period (mean difference, 0.04 [95% CI, −0.20 to 0.27] days). There were no statistical differences between the intervention and baseline for length of hospital stay (mean [SD], 5.4 [4.8] vs 5.4 [5.0] days; mean difference 0.11 [95% CI, −0.12 to 0.33] days) or hospital readmission within 30 days (OR, 1.02 [95% CI, 0.88 to 1.18]). Other secondary outcomes are shown in Table 3.

During the baseline and intervention periods, 4186 patients (29.3%) and 2085 patients (27.6%), respectively, had 1 of the common infections listed as a discharge diagnosis. Total antibiotic duration, which included both inpatient and postdischarge therapy, did not differ between the baseline and intervention periods for these individual infection types except for intra-abdominal infections (Table 4).

**Table 4.** Total Antibiotic Duration for Specific Infections Across Participating Units From 10 Hospitals During Baseline and Intervention Periods^a^

Infection	No.	Total antibiotic duration, mean (SD), d			*P* value
		Baseline	Intervention	Mean (95% CI) difference	
Respiratory^b^	2681	6.3 (5.3)	6.3 (5.6)	−0.02 (−0.47 to 0.42)	.92
Skin and soft tissue	1423	10.5 (6.5)	11.1 (7.7)	0.62 (−0.13 to 1.38)	.12
Urinary tract^c^	1409	8.7 (6.0)	9.1 (6.2)	0.43 (−0.22 to 1.09)	.20
Intra-abdominal and biliary	672	11.3 (8.6)	9.3 (7.7)	−1.98 (−3.23 to −0.73)	.002

^a^ Total antibiotic duration (ie, inpatient plus postdischarge) was evaluated only for patients who were prescribed antibiotics and had a qualifying discharge diagnostic code. All differences were calculated as intervention minus baseline. Differences were assessed using 2-sample independent *t* tests with pooled variance, except intra-abdominal and biliaryinfections.

^b^ Respiratory infections include acute exacerbations of chronic obstructive pulmonary disease and pneumonia without abscess or empyema.

^c^ Urinary tract infections include both uncomplicated and complicated infections.

Based on manual EHR review of 434 cases, optimal antibiotic prescribing at discharge increased from 122 of 264 cases (46.2%) at baseline to 100 of 170 cases (58.8%) during the intervention period. In an adjusted analysis, the odds of receiving optimal antibiotics at discharge were significantly higher during the intervention period relative to the baseline period (OR, 1.61 [95% CI, 1.08-2.40]). eTables 4 and 5 in Supplement 2 summarize the EHR review findings.

#### Reach

There were an estimated 721 frontline prescribers who worked on a participating unit during an intervention period, and 275 frontline prescribers (38.1%) received feedback at least once as part of the discharge-focused audit and feedback process. At the hospital-level, the median (IQR) reach was 44.0% (34.1%-54.5%).

#### Implementation

##### Acceptability

Of 112 frontline prescribers sent a postintervention electronic survey, 40 (35.7%) responded; 4 reported never receiving feedback and were therefore not asked to complete any more survey questions. Only 1 site had no respondents. Nearly all respondents (34 of 36 [94.4%]) stated that the AS team’s discharge-focused initiative improved antibiotic prescribing at discharge, and 31 of 34 respondents (88.6%) agreed that the discharge-focused intervention should continue (eTable 6 in Supplement 2).

##### Feasibility

All AS teams were able to implement the intervention. In the aforementioned electronic survey, 29 of 36 respondents (80.6%) reported that the recommendations of the AS team about antibiotic prescribing were usually or always given in time for changes to be made prior to discharge (eTable 6 in Supplement 2).

##### Fidelity

At the hospital level, the median (IQR) number of days per week on which audits were performed was 4.6 (4.1-4.8) days. Eight AS teams (80.0%) did at least 1 audit every week that they were randomized to the intervention, and 7 AS teams (70.0%) gave feedback at least 1 time every week.

##### Time Commitment

At the hospital level, the median (IQR) time commitment from AS pharmacists was 1.9 (0.7-2.4) hours per week; the median (IQR) time commitment from clinical pharmacists was 0.8 (0.4-1.3) hours per week. AS physician effort was minimal (eTable 3 in Supplement 2).

#### Maintenance

Only 1 site (10.0%) was still conducting discharge-focused audit and feedback activities 6 months after the trial ended. Six sites (60.0%) stated that they were still performing some type of AS focused on discharge, but these sites were no longer prospectively auditing patients receiving antibiotics who were approaching discharge. Instead, 4 AS teams were making recommendations on antibiotic use, including postdischarge use, earlier during the hospital stay; 1 AS team was discussing postdischarge antibiotics at multidisciplinary rounds prior to discharge; and 1 hospital empowered clinical pharmacists embedded within the hospital medicine teams to make recommendations at the point of discharge.

## Discussion

In this stepped-wedge cluster-randomized clinical trial across 10 hospitals, a discharge-focused audit and feedback process did not decrease overall antibiotic use at hospital discharge, but it did improve optimal antibiotic prescribing in a group of patients with common and uncomplicated diagnoses. Frontline prescribers who responded to our survey found the AS team’s feedback helpful and wanted the discharge-focused process to continue.

There could be several reasons why the intervention did not decrease overall postdischarge antibiotic use. First, because all hospitals had active inpatient AS strategies in place prior to the intervention, it is possible that AS strategies applied earlier in the hospital stay decreased the need for antibiotic optimization as the patient approached hospital discharge. However, a large proportion of patients who met our carefully defined criteria for manual EHR reviews still received suboptimal antibiotic therapy at discharge. Second, many discharging patients may not have been touched by the intervention, either because their infection was too complicated or because an opportunity to optimize therapy was not addressed prior to discharge. Based on our survey, frontline prescribers believed that 1 of every 5 AS team recommendations was not made in a timely manner. Finally, frontline prescribers may have disagreed with the AS team’s input or simply forgotten to apply it when entering their discharge antibiotic orders.

The intervention did increase optimal antibiotic prescribing at hospital discharge in a highly selected group of patients. But given the small magnitude of improvement (12.6%), it may be difficult to justify continuing the discharge-focused audit and feedback process. In fact, all but 1 hospital stopped performing it when the trial ended.

Our findings indicate that the auditing process was fairly inefficient in identifying opportunities to shorten antibiotic duration at discharge. While approximately one-quarter of all audits led to a recommendation from the AS team, only 44% of these recommendations were about stopping antibiotics or modifying duration of therapy. In other words, roughly 10% of all patients audited led to feedback on modifying the planned duration of postdischarge antibiotic therapy. Prior studies have found that discharge-focused audits find opportunities to give feedback in only 10% to 25% of all cases.^16,17,18^ Overall, these findings highlight a need for automated strategies to identify cases in which AS team input would be of greatest benefit.

Our findings differ from a multicenter, nonrandomized stepped-wedge trial that more than doubled optimal antibiotic prescribing at discharge after implementation of a discharge-focused bundled intervention.^12^ One key difference between these studies is that the prior trial had the clinical pharmacist enter the discharge antibiotic order for the frontline prescribers to cosign. This additional nudge, which was only performed after obtaining the prescriber’s buy-in, likely ensured that the antibiotic prescribed at discharge was optimized in terms of its selection, dose, and duration. In addition, the prior trial only measured optimal antibiotic prescribing in selected patients, so its findings may be less generalizable than our study, which measured postdischarge antibiotic use in all patients discharged to the community.

### Limitations

There are several limitations to our study. First, some hospitals only performed the intervention for a few months; thus, there were limited opportunities to optimize the audit and feedback process. While our study design was chosen to answer the research question as efficiently as possible, a longer intervention period may have demonstrated more of an effect. Second, we were unable to accurately measure how often the stewardship team’s recommendations were actually accepted; thus, it is possible that the acceptance rate was much lower than what was perceived. Third, our primary outcome was postdischarge antibiotic frequency and duration, which did not account for whether there were improvements in antibiotic selection. Fourth, we suspect there was variation across sites in how the audit and feedback process was implemented, and it is unclear how this variation affected outcomes. Fifth, we were unable to gather data on postdischarge antibiotics prescribed to patients transferred to skilled nursing facilities or discharged with outpatient parenteral antibiotic therapy. Based on our prior work, these patients could have accounted for a substantial proportion of all antibiotics prescribed at discharge.^19^ Sixth, our manual EHR reviews excluded approximately half of all patients with one of the common infection types, largely because their infections were too complicated. Shortening antibiotic duration may be easier in these less complicated cases that represent only a small proportion of all antibiotic-treated patients. In addition, our findings are only generalizable to similar types of hospitals with similar inpatient stewardship activities in place.

## Conclusions

In this stepped-wedge cluster-randomized clinical trial across 10 hospitals, a discharge-focused prospective audit and feedback process did not decrease overall antibiotic use at hospital discharge. Other strategies to address antibiotic overuse at discharge are needed.

## Notes

Supplement 1.
Trial Protocol

Supplement 2. **eAppendix.** Protocol for manual chart reviews **eTable 1.** Timeline of study pre-implementation and implementation activities **eTable 2.** Processes used at each hospital for identifying patients who would possibly be discharged on oral antibiotics and for giving feedback to prescribers **eTable 3.** Fidelity to and time commitment of the intervention, based on weekly surveys completed by each site during the intervention period **eTable 4.** Number of cases excluded and included from manual chart reviews across participating units from the 10 hospitals **eTable 5.** Findings from manual chart reviews of 434 patients across participating units from the 10 hospitals during the baseline and intervention periods **eTable 6.** Findings from a post-intervention electronic survey of frontline antibiotic prescribers at all ten participating sites

Supplement 3.
Data Sharing Statement
