# Supplementary material for: Prospective Audit and Feedback by Antibiotic Stewardship Teams to Reduce Antibiotic Overuse at Hospital Discharge: A Stepped-Wedge Cluster-Randomized Clinical Trial
Source: JAMA Netw Open. 2026 Jan 9;9(1):e2549655. doi: 10.1001/jamanetworkopen.2025.49655 (PMC12789953; doi:10.1001/jamanetworkopen.2025.49655)
Supplement: Supplement 1. — Trial Protocol [file jamanetwopen-e2549655-s001.pdf]

Improving post-discharge antimicrobial use: a multicenter stepped wedge trial

Protocol date March 24, 2023

Version 1.5

Principal investigator: Daniel Livorsi, MD, MSc

Data analyst/statistician: Amy O'Shea, PhD

BACKGROUND

Antimicrobial stewardship programs (ASPs) often restrict their activities to inpatient antimicrobial-prescribing. However, at least 40% of all antimicrobial exposure associated with an acute-care hospital stay is prescribed at the time of hospital discharge (i.e., post-discharge).[1-3] Post-discharge antimicrobials mediate clinical outcomes after discharge and may facilitate the spread of antimicrobial resistance.

Several studies have shown that post-discharge antimicrobial use is often inappropriate. We used national data to show that 61% of fluoroquinolone treatment days were prescribed at hospital discharge; manual chart reviews at 9 hospitals found that 40% of these post-discharge fluoroquinolone prescriptions were either unnecessary or sub-optimal.[4] Other studies have found that 53-79% of all post-discharge antimicrobials are either unnecessary or sub-optimal.[5-7]

Post-discharge antimicrobials are an important target for antimicrobial stewardship. However, inpatient stewardship metrics do not capture post-discharge antimicrobials and ASPs frequently do not evaluate these prescriptions. A 2016 Veterans Health Administration (VHA) survey found that less than 50% of hospitals routinely reviewed targeted antimicrobials at discharge.[8] According to a 2016 survey in Michigan, only 17% of 48 hospitals had a process for reviewing outpatient antimicrobial orders at discharge.[9] Nearly all hospitals responding to these two surveys reported that they had an inpatient ASP.

We currently do not know how inpatient ASP resources can be effectively leveraged to improve post-discharge antimicrobial use. Several randomized-controlled trials have demonstrated that post-prescription audit-and-review (PPR) can reduce and improve inpatient antimicrobial use, but these studies did not measure the effect of PPR on post-discharge antimicrobial use.[10-12] Two single-center non-randomized studies, including a study by our team of investigators, showed that inpatient PPR can decrease and improve post-discharge antimicrobial use.[1, 13] However, these findings may not be generalizable to other settings.

If the goal is to improve post-discharge antimicrobial use, a potentially effective strategy may be a PPR process focused solely on prescriptions for patients who will soon be discharged. However, such a PPR intervention would require an innovative approach, as ASPs typically do not know when patients will be discharged.

METHODS

<u>Trial design:</u>

We will perform a stepped-wedge cluster-randomized trial to assess the effectiveness of discharge- post-prescription audit-and-review (i.e. discharge-PPR) in safely reducing post-discharge antimicrobial use.

There are ten sites taking part in this study. Based on the stepped wedge study design, randomization will be performed at the site-level with all eligible patients having access to the intervention once a site has entered the intervention phase. Each facility will begin in control status with one facility crossing into the intervention every two weeks following a 24-week baseline period. A simple randomization scheme using a random number generator will be used a priori to determine the order of implementation. Once clinical rollout is complete, all sites will continue to perform the intervention for another 2.5-3 months. No site will perform the intervention for longer than 6 months.

Participants: We will enroll up to 9 antimicrobial stewardship teams and their respective hospital sites (n=10). Note that one stewardship team covers two hospitals.

Inclusion criteria: An acute-care hospital with an antimicrobial stewardship team

Exclusion criteria: A discharge-focused PPR process is already in place or will be implemented soon

Method of recruitment: We will recruit sites through the EpiCenter's national conference calls and known professional contacts of the study PI.

Interventions:

Pre-intervention activities: All sites must identify a site principal investigator (PI), i.e. a physician who preferably has experience with antimicrobial stewardship. Each site must also identify a study pharmacist. Ideally, the study pharmacist will already be part of stewardship activities.

- *Baseline survey of ASP activities*: All participating hospitals will complete an electronic survey about their baseline ASP activities.
- *Process development*: Before the intervention, the pharmacist and site PI will develop a process for identifying eligible patients for discharge-PPR audits; this process may include using information technology tools and collaborating with case managers or discharge coordinators.
- *Collaborative learning calls*: Nine months before the intervention begins, the study coordinator will start organizing collaborative learning calls for the study PI (D. Livorsi) and the study personnel (site PI and pharmacist) at all participating sites. During these monthly calls, sites will share their experience developing a process to identify patients who will be discharged within 48 hours. In addition, participants will review the audit-and-feedback literature and their own personal experience with stewardship to facilitate discussion about the optimal approach to discharge-PPR .[13-15]

Intervention activities:

During the intervention, a study team member (either a physician or pharmacist) will perform discharge-PPR every weekday for patients on select medical services who are receiving an antimicrobial and whose discharge is anticipated within the next 48 hours. The number of services selected for PPR will be aimed at providing approximately 200 discharges per month per facility.

The site PI will provide physician-support for these PPR activities. Discharge-PPR audits will focus on optimizing antimicrobial selection, dose, and duration before discharge. The pharmacist will communicate all recommendations resulting from the audit to the primary prescribers. Site PIs and pharmacists will also participate in the collaborative learning calls every month during their sites' intervention periods.

All hospitals may continue their usual stewardship activities during the trial. However, hospitals will be strongly encouraged to not start any other new stewardship interventions targeting antimicrobial-prescribing at discharge.

Further description of discharge-PPR process:

- Content: Real-time feedback on the selection, dose, and duration of antimicrobials prescribed at hospital discharge

- Delivery method: Feedback will be preferably delivered in-person or by telephone. Electronic, asynchronous communication may also be used.

- Unit of delivery: The feedback will be given to an inpatient provider directly caring for the patient who is receiving antimicrobials.

- Deliverer: Feedback will be delivered by the participating stewardship teams or involved extenders.

- Setting: The intervention will be delivered on the acute-care hospital units before patients receiving antimicrobials are discharged.

- Exposure quantity and duration: Feedback will be given during weekday working hours.

- Time span: 2-6 months, depending on when a site is randomized to the intervention

- Activities to increase compliance or adherence: Baseline education about duration of antimicrobial therapy for common infections treated in hospitalized patients.

Objectives:

AIM 1: Evaluate whether discharge post-prescription audit-and-review (PPR) can safely reduce post-discharge antimicrobial use across participating hospitals.
Hypothesis 1: The discharge audit-and-feedback intervention will reduce post-discharge antimicrobial use without adversely affecting patient safety.

AIM 2: Through monthly collaborative learning calls with participating sites, determine barriers and facilitators encountered by antimicrobial stewardship teams in their implementation of the discharge-PPR process.

Outcomes:

We will use the RE-AIM framework (page 6) to assess our pilot trial. Clinical outcomes are listed below. Data for all clinical outcomes will be extracted from the respective data systems of each participating site. All data will be submitted to the project PI for final data analysis.

Primary outcome
The primary outcome will be post-discharge antimicrobial length of therapy (LOT) per 100 admissions. This outcome will be collected every two weeks during the study period for all services or wards participating in the project.

Secondary outcomes

Additional outcomes will include inpatient antimicrobial use (inpatient LOT per 100 admissions), length of stay, and 30-day hospital re-admission rates. These secondary outcomes will only be measured for patients who were discharged from a participating service or ward.

Process measures: Each day, the study pharmacist will record the number of patients audited during discharge-PPR, the number of PPR recommendations communicated to providers, and the number of recommendations that were accepted.

Implementation outcomes: We will use the RE-AIM framework to assess our intervention. Feasibility will be evaluated on 2 domains: 1) the development of a local process to identify patients who are on antimicrobials within 48 hours of hospital discharge, and 2) the initiation of the discharge-PPR intervention. To further understand the implementation process, the study coordinator will take notes during the collaborative learning calls. We will also record these calls. We will perform a rapid template analysis to describe each site's implementation process, including barriers and facilitators to successful implementation.[16] Through a weekly survey, we will also assess each site's fidelity to the discharge-PPR process and the cost (i.e. time) of the intervention. Through a post-intervention electronic survey, we will assess the acceptability of discharge-PPR to frontline prescribers.

Sample Size:

We will calculate the average post-discharge LOT per 100 admissions every two weeks and will include 10 sites. For these power calculations, we assume: 1) an intra-cluster correlation of 0.75; 2) an effect size of 10% from the baseline average post-discharge LOT per 100 admission of 135; 3) 100 discharges per site every two weeks; ; 4) a cluster-level standard deviation of 3.4; 5) a Type I error rate of 5%, and 6) a constant treatment effect regardless of exposure time. Given these assumptions, under a stepped wedge study design with 1) each step lasting 2 weeks (24 measurements/site over 48 weeks total); 2) all 10 facilities starting in control status and 1 facility crossing into intervention status every 2 weeks following a baseline period of 24 weeks and ending with all facilities in intervention status in the final two-week block, we have more than 90% power to determine a statistical difference when discharge-PPR is used.

Blinding: None

Unit of Analysis: Each medical center

Statistical Methods: We will evaluate the effectiveness of implementing a discharge-focused process for post-prescription audit-and-review (PPR) via a generalized linear mixed model with identity link. The primary outcome will be post-discharge length of therapy (LOT) per 100 admissions. This model will include step and intervention indicators as fixed effects and a random intercept for cluster to account for facility-level factors. This model structure can provide proper weighting when cluster sizes vary and account for repeated measurements over time. Confounding variables (select covariates that may be associated with optimal antimicrobial prescribing) will be adjusted for statistically by inclusion in the model framework.

Applying the RE-AIM implementation framework to our evaluation of this pilot study's findings

Dimension	Definition[1]	Measurement
**Reach**	The absolute number, proportion and representativeness of individuals who are willing to participate in a given initiative or program, and reasons why they do or do not participate<br>Who actually participates or is exposed to the initiative?	Monitor the number of prescribers at each site who receive feedback.
**Effectiveness**	The effect of an intervention on important individual outcomes, such as clinical events, quality of life, and economic outcomes	See primary and secondary outcomes, as listed above
**Adoption**	The absolute number, proportion and representativeness of settings and agents who are willing to start a program and why they are willing to start it<br>Where is the program applied and who applied it?	Describe the location and complexity of the intervention sites. During the introductory call, ask the teams at the intervention sites why they want to participate.
**Implementation**	Fidelity to the key elements of an evidence-base practice, including consistency of delivery as intended and the time and cost of the program<br><br>How consistently was the program delivered? How was it adapted to each setting? How much did it cost? Why did it achieve the observed result?	Measure fidelity by tracking the number of audits performed and recommendations made.<br><br>Measure acceptability with a post-intervention survey of frontline prescribers.<br><br>Measure implementation cost by having the project team track the time committed to project activities on a weekly basis.
**Maintenance**	The extent to which a program or policy becomes institutionalized or part of the routine organizational practices and policies.<br>*How long are the results of the program or policy sustained?*	Assess whether sites are still performing discharge audit-and-feedback 6 months after the project ends

1. All definitions are taken from https://www.re-aim.org

References:

1. Tamma PD, Avdic E, Keenan JF, et al. What is the More Effective Antibiotic Stewardship Intervention: Pre-Prescription Authorization or Post-Prescription Review with Feedback? *Clin Infect Dis*. Dec 07 2016;doi:10.1093/cid/ciw780
2. Dyer AP, Dodds Ashley E, Anderson DJ, et al. Total duration of antimicrobial therapy resulting from inpatient hospitalization. *Infect Control Hosp Epidemiol*. May 28 2019:1-8. doi:10.1017/ice.2019.118
3. Feller J, Lund BC, Perencevich EN, et al. Post-discharge oral antimicrobial use among hospitalized patients across an integrated national healthcare network. *Clin Microbiol Infect*. Oct 7 2019;doi:10.1016/j.cmi.2019.09.016
4. Suzuki H, Perencevich EN, Alexander B, et al. Inpatient Fluoroquinolone Stewardship Improves the Quantity and Quality of Fluoroquinolone-prescribing at Hospital Discharge: A Retrospective Analysis among 122 Veterans Health Administration Hospitals. *Clin Infect Dis*. Sep 28 2019;doi:10.1093/cid/ciz967
5. Chavada R, Davey J, O'Connor L, Tong D. 'Careful goodbye at the door': is there role for antimicrobial stewardship interventions for antimicrobial therapy prescribed on hospital discharge? *BMC Infect Dis*. May 16 2018;18(1):225. doi:10.1186/s12879-018-3147-0
6. Yogo N, Shihadeh K, Young H, et al. Intervention to Reduce Broad-Spectrum Antibiotics and Treatment Durations Prescribed at the Time of Hospital Discharge: A Novel Stewardship Approach. *Infect Control Hosp Epidemiol*. May 2017;38(5):534-541. doi:10.1017/ice.2017.10
7. Scarpato SJ, Timko DR, Cluzet VC, et al. An Evaluation of Antibiotic Prescribing Practices Upon Hospital Discharge. *Infect Control Hosp Epidemiol*. Mar 2017;38(3):353-355. doi:10.1017/ice.2016.276
8. 2015 Survey of Antimicrobial Stewardship in VHA (2016).
9. Vaughn VM PL, Flanders, SA, Malani AN, Patel T, et al. A Deeper Dive into Antibiotic Stewardship Needs: A Multi-Hospital Survey. *Open Forum Infect Dis*. 2020;
10. Camins BC, King MD, Wells JB, et al. Impact of an antimicrobial utilization program on antimicrobial use at a large teaching hospital: a randomized controlled trial. *Infect Control Hosp Epidemiol*. Oct 2009;30(10):931-8. doi:10.1086/605924
11. Lesprit P, Landelle C, Brun-Buisson C. Clinical impact of unsolicited post-prescription antibiotic review in surgical and medical wards: a randomized controlled trial. *Clin Microbiol Infect*. Feb 2013;19(2):E91-7. doi:10.1111/1469-0691.12062
12. Stenehjem E, Hersh AL, Buckel WR, et al. Impact of Implementing Antibiotic Stewardship Programs in 15 Small Hospitals: A Cluster-Randomized Intervention. *Clin Infect Dis*. Aug 1 2018;67(4):525-532. doi:10.1093/cid/ciy155
13. Griebel ME, Heintz B, Alexander B, Egge J, Goto M, Livorsi DJ. Understanding changes in the standardized antimicrobial administration ratio for total antimicrobial use after implementation of prospective audit and feedback. *Infect Control Hosp Epidemiol*. Dec 2018;39(12):1476-1479. doi:10.1017/ice.2018.248
14. Brehaut JC, Colquhoun HL, Eva KW, et al. Practice Feedback Interventions: 15 Suggestions for Optimizing Effectiveness. *Ann Intern Med*. Mar 15 2016;164(6):435-41. doi:10.7326/M15-2248
15. Ivers N, Jamtvedt G, Flottorp S, et al. Audit and feedback: effects on professional practice and healthcare outcomes. *Cochrane Database Syst Rev*. Jun 13 2012;(6):CD000259. doi:10.1002/14651858.CD000259.pub3
16. N K. Using templates in the thematic analysis of text. In: Symon CCaG, ed. *Essential Guide to Qualitative Methods in Organizational Research*. Sage Publications; 2004:256-70.
17. Proctor E, Silmere H, Raghavan R, et al. Outcomes for implementation research: conceptual distinctions, measurement challenges, and research agenda. *Adm Policy Ment Health*. Mar 2011;38(2):65-76. doi:10.1007/s10488-010-0319-7
