# Supplementary material for: Prospective Audit and Feedback by Antibiotic Stewardship Teams to Reduce Antibiotic Overuse at Hospital Discharge: A Stepped-Wedge Cluster-Randomized Clinical Trial
Source: JAMA Netw Open. 2026 Jan 9;9(1):e2549655. doi: 10.1001/jamanetworkopen.2025.49655 (PMC12789953; doi:10.1001/jamanetworkopen.2025.49655)
Supplement: Supplement 2. — eAppendix. Protocol for manual chart reviews eTable 1. Timeline of study pre-implementation and implementation activities eTable 2. Processes used at each hospital for identifying patients who would possibly be discharged on oral antibiotics and for giving feedback to prescribers eTable 3. Fidelity to and time commitment of the intervention, based on weekly surveys completed by each site during the intervention period eTable 4. Number of cases excluded and included from manual chart reviews across participating units from the 10 hospitals eTable 5. Findings from manual chart reviews of 434 patients across participating units from the 10 hospitals during the baseline and intervention periods eTable 6. Findings from a post-intervention electronic survey of frontline antibiotic prescribers at all ten participating sites [file jamanetwopen-e2549655-s002.pdf]

Supplementary Online Content

Livorsi DJ, Thompson AM, Green MS, et al. Prospective audit and feedback by antibiotic stewardship teams to reduce antibiotic overuse at hospital discharge: a stepped-wedge cluster-randomized clinical trial. *JAMA Netw Open*. 2026;9(1):e2549655.
doi:10.1001/jamanetworkopen.2025.49655

eAppendix. Protocol for manual chart reviews

eTable 1. Timeline of study pre-implementation and implementation activities

eTable 2. Processes used at each hospital for identifying patients who would possibly be discharged on oral antibiotics and for giving feedback to prescribers

eTable 3. Fidelity to and time commitment of the intervention, based on weekly surveys completed by each site during the intervention period

eTable 4. Number of cases excluded and included from manual chart reviews across participating units from the 10 hospitals

eTable 5. Findings from manual chart reviews of 434 patients across participating units from the 10 hospitals during the baseline and intervention periods

eTable 6. Findings from a post-intervention electronic survey of frontline antibiotic prescribers at all ten participating sites

This supplementary material has been provided by the authors to give readers additional information about their work.

© 2026 Livorsi DJ et al. *JAMA Network Open*.

eAppendix. Protocol for manual chart reviews

Inclusion criteria:

- Discharged from a unit/ward participating in the intervention trial.
- Discharged on a weekday
- Received inpatient antibiotics on the day before discharge and/or the day of discharge
- Discharge diagnosis of acute exacerbation of chronic obstructive pulmonary disease (COPD), uncomplicated pneumonia, urinary tract infection (UTI, complicated or uncomplicated), skin and soft tissue infection (SSTI), intra-abdominal infection or biliary tract infection
- Prescribed outpatient oral antibiotics at the time of discharge

Exclusion criteria:

- Age < 18
- Antibiotic duration (inpatient + post-discharge) < 3 days
- Died in the hospital
- Discharged against medical advice
- Transferred to another care facility, including hospice, hospital, nursing home, or other non-acute care location
- Discharge diagnostic code for endocarditis, meningitis, osteo-articular infection, complicated pneumonia,

Admissions should be randomly selected from the baseline or intervention periods:

Sampling of cases to review:

- Admissions were randomly selected from the baseline or intervention periods from each hospital.
- For each hospital, randomly selected cases were reviewed until 30 cases were included for the baseline period and 20 for the intervention period. However, at small sites or sites with short intervention periods, it was sometimes not possible to find enough eligible cases, either due to low patient-volumes and/or the high exclusion rate after applying the above criteria.
- Each included case was reviewed by two independent reviewers. If the two reviewers had a discordant assessment, the two reviewers met face-to-face to reach consensus.

Narrow versus broad-spectrum categorization of oral antibiotics
https://academic.oup.com/cid/article/75/4/567/6463001?login=true

NARROW SPECTRUM (DASC score ≤ 8)	BROAD SPECTRUM (DASC score ≥ 9)
Amoxicillin<br>Amoxicillin/clavulanate<br>Azithromycin<br>Cefadroxil<br>Cefaclor<br>Cefdinir<br>Cefpodoxime<br>Cefuroxime<br>Cephalexin<br>Clarithromycin<br>Clindamycin<br>Dicloxacillin	Ciprofloxacin<br>Delafloxacin<br>Levofloxacin<br>Moxifloxacin<br>Omadacycline

© 2026 Livorsi DJ et al. *JAMA Network Open*.

Doxycycline<br>Fosfomycin<br>Linezolid<br>Metronidazole<br>Minocycline<br>Nitrofurantoin<br>Penicillin<br>TMP/SMX	

LOWER RESPIRATORY TRACT INFECTION

1. **Apply manual exclusion criteria**
   a. Diagnostic code was wrong, and there is no infection
   b. Solid organ transplant
   c. Neutropenia (ANC < 500)
   d. Cystic fibrosis
   e. HIV with CD4 count < 200
   f. Primary immunodeficiency
   g. Admitted from an outside acute-care hospital <u>and</u> antibiotic therapy prior to transfer is not known
   h. Discharged with IV antibiotics
   i. Empyema, complicated parapneumonic effusion, lung abscess, post-obstructive pneumonia
   j. Pneumonia due to a mycobacterial or fungal pathogen, Actinomyces, Nocardia, or *Rhodococcus*
   k. An infection other than pneumonia was also being treated with antibiotics
   l. Bloodstream infection due to *Candida*, *Enterococcus* species or *Staphylococcus aureus*
2. **Classify infection based on documented signs/symptoms, laboratory data, and radiologic studies**
   a. Community-acquired pneumonia: pneumonia acquired outside the hospital or < 48 hours after admission
   b. Hospital-acquired pneumonia: occurs ≥48 hours after admission & was not incubating at the time of admission
   c. Acute exacerbation (AE) of COPD that warrants antibiotics:
      i. purulent sputum + either increase in dyspnea or increase in sputum volume; OR
      ii. patient required mechanical ventilation (invasive or noninvasive)
   d. Viral respiratory process
   e. No infection
3. **Assess <u>antibiotic selection</u> at discharge, based on infection type, microbiology results, and local guidelines**
   a. *Unnecessary*: antibiotic therapy was prescribed at discharge but not needed either because the patient had already completed a full course or did not have a bacterial infection
   b. *Inappropriate*: the prescribed antibiotic regimen at discharge was not active against the isolated or suspected pathogen(s)
   c. *Sub-optimal*: the use of a broad-spectrum antibiotic agent at discharge when a narrow agent would have sufficed, based on microbiologic testing and known allergies. Examples include:
      i. Pneumonia: prescribing a quinolone when micro testing revealed a pathogen treatable with beta-lactams
      ii. COPD-E: prescribing levo when there are no risk factors for Pseudomonas aeruginosa colonization
   d. *Optimal*: None of the above definitions (a-c) are met.
      - If a pathogen is identified, an optimal regimen at discharge would be active against the pathogen and, if possible, narrow-spectrum. If a broad agent was used when a narrow

© 2026 Livorsi DJ et al. *JAMA Network Open*.

one would have been active and tolerated by the patient (based on their known allergy history), then choose “sub-optimal.”

- If treatment is empiric, then use the following guidance to assess antibiotic use at discharge:

i. *AE of COPD*: amox/clav, azithromycin, cefuroxime, cefdinir, cefpodoxime, doxycycline (levo OK if prior or current respiratory colonization with Pseudomonas aeruginosa)

ii. *CAP*: amox/clav ± azithromycin; cefuroxime/cefdinir/cefpodoxime ± azithromycin, levo- or moxifloxacin

1. OK to substitute doxycycline for azithromycin based on side effect profile
2. OK to forego atypical coverage if atypicals are adequately ruled-out
3. Please consider MRSA and Pseudomonas risk factors, as outlined in ATS/IDSA CAP guidelines

iii. *HAP*: If MRSA nares (-), options include amox/clav, cefuroxime/cefdinir/cefpodoxime, levo- or moxi. Please refer to ATS/IDSA guidelines on HAP for further guidance on empiric therapy.

4. **Assess prescribed antibiotic duration at discharge, based on infection type, clinical response, micro results**

**Account for inpatient duration of therapy when assessing duration prescribed at discharge*

Acute exacerbation of COPD that meets criteria for antibiotics: 5 days, or 1.5 grams of azithromycin over 3 days

Community-acquired pneumonia: 5 days, assuming patient has been afebrile for 48 hrs, breathing without supplemental oxygen (unless required previously), and had no more than one clinical instability factor (defined as heart rate >100 beats/minute, respiratory rate >24 breaths/minute, and SBP ≤90 mmHg)

- Antibiotic duration can be longer (e.g. 7 days) if MRSA, Pseudomonas aeruginosa, or Legionella are isolated
- OK to give antibiotic duration of 3 days, assuming clinical stability reached at 72 hours: temp ≤ 100ºF, HR < 100/minute, RR< 24/minute, O2 saturation ≥ 90%, SBP ≥ 90 mm Hg, normal mental status, and no Legionella, MRSA, or Pseudomonas

Hospital-acquired pneumonia: 7 days, but 5-day course is reasonable if above clinical stability criteria were met and organism is not MRSA or Pseudomonas

Use the following criteria when assessing antibiotic duration:

a. If antibiotic selection was deemed “unnecessary” or “inappropriate,” then choose “Not applicable.”
b. If duration is +/- 1 day of the recommended duration, then classify as “optimal”
c. If duration is ≥ 2 days of the recommended duration, then classify as “too long”
d. If duration is < 2 days of the recommended duration, then classify as “too short”

URINARY TRACT INFECTION

1. **Apply manual exclusion criteria**

a. Diagnostic code was wrong, and there is no infection
b. Solid organ transplant
c. Neutropenia (ANC < 500)
d. Admitted from an outside acute-care hospital and antibiotic therapy prior to transfer is not known
e. Discharged with IV antibiotics

© 2026 Livorsi DJ et al. *JAMA Network Open*.

f. An infection outside the urinary tract that was also being treated with antibiotics
g. Bloodstream infection due to *Candida*, *Enterococcus* species or *Staphylococcus aureus*
h. Complicated urinary tract infection, such as peri-nephric abscess, acute bacterial prostatitis or epididymo-orchitis

2. **Review the urinalysis and urine culture**
   a. If the urinalysis showed < 5 WBCs per high-power field OR a urine culture (obtained prior to antibiotics) was negative or only grew skin flora OR signs/symptoms attributable to a UTI were not present, then choose "UTI was not present"
   b. If the urinalysis showed ≥ 5 WBCs per high-power field, urine culture grew a likely pathogen, and signs/symptoms of a UTI were documented, then choose "UTI was present."
      i. If the urine culture was "contaminated" or was negative because antibiotics had already been started prior to collection BUT the patient meets all other criteria under (b), include the patient in this category.
   c. If there is no urinalysis, no urine culture, or no documentation of either the presence/absence of UTI symptoms or urine studies are not interpretable, then exclude the case because "Data is incomplete",
3. **Classify infection based on documented signs/symptoms, laboratory data, and radiologic studies**
   a. UTI is present
      i. Cystitis without catheter: pyuria (≥5 WBCS per HPF), positive urine culture
      ii. Cystitis with catheter
      iii. Pyelonephritis (or bacteremic UTI)
   b. UTI is not present
      i. Asymptomatic bacteriuria/pyuria
      ii. No pyuria and/or no bacteriuria
   c. Unclear if UTI is present or absent
4. **Assess <u>antibiotic selection</u> at discharge, based on infection type and microbiology results**
   a. *Unnecessary*: antibiotic therapy was prescribed at discharge but not needed either because the patient had already completed a full course or did not have a bacterial infection
   b. *Inappropriate:* the prescribed antibiotic at discharge was not active against the isolated pathogen(s) or, in the absence of a positive urine culture, was not a recommended empiric agent (either based on below guidance or prior urine cultures)
   c. *Sub-optimal*: the use of a broad-spectrum antibiotic agent for cystitis when a narrow agent would have sufficed, based on microbiologic testing and known allergy history.
   For pyelonephritis, fluoroquinolones should NOT be considered sub-optimal.*Optimal*: none of the above definitions (a-c) are met AND antibiotics were active against the isolated or suspected pathogens

   Empiric antibiotic options

   **The below table is not comprehensive. Use your clinical judgement if the prescribed agent isn't on this list. Prior urine cultures may also be informative in guiding empiric therapy.*

**Cystitis**	**Antibiotic options**
No indwelling catheter, no recent antibiotic exposure	Nitrofurantoin, cephalexin, TMP/SMX
Indwelling catheter or recent antibiotic exposure	Cefdinir, ciprofloxacin
**Pyelonephritis**	Ciprofloxacin, TMP/SMX

5. **Assess prescribed <u>antibiotic duration</u> at discharge, based on infection type, clinical response, micro results**

**Account for inpatient duration of therapy when assessing duration prescribed at discharge*

© 2026 Livorsi DJ et al. *JAMA Network Open*.

Cystitis: antibiotic duration=7 days except in cases of uncomplicated cystitis, defined as a lower urinary tract infection in a healthy, non-pregnant, pre-menopausal woman with no abnormal urinary tract: 3 days for TMP/SMX and ciprofloxacin; 5 days for nitrofurantoin and oral beta-lactams

Pyelonephritis: antibiotic duration=7 days if patient responds to therapy and entire course is IV or transitioned to oral fluoroquinolone or TMP-SMX; if therapy completed with oral beta lactam, then treat for 10 total days.

Use the following criteria when assessing antibiotic duration:

a. If antibiotic selection was deemed "unnecessary" or "inappropriate," then choose "Not applicable."
b. If duration is +/- 1 day of the recommended duration, then classify as "optimal"
c. If duration is ≥ 2 days of the recommended duration, then classify as "too long"
d. If duration is < 2 days of the recommended duration, then classify as "too short"

SKIN AND SOFT TISSUE INFECTION

1. **Apply manual exclusion criteria**
    a. Diagnostic code was wrong, and there is no infection
    b. Solid organ transplant
    c. Neutropenia (ANC < 500)
    d. Admitted from an outside hospital and antibiotic therapy prior to transfer is not known
    e. Discharged with IV antibiotics
    f. Any infection beyond the skin and soft tissue that was being treated with antibiotics, including:
        i. Infection of adjacent structures (e.g., contiguous osteomyelitis associated with a wound), or
        ii. An entirely different body site (e.g., pneumonia)
    g. Bloodstream infection due to *Candida*, *Enterococcus* species or *Staphylococcus aureus*
    h. Complicating factors, such as:
        i. Bite wound
        ii. Gangrene associated with PVD
        iii. Necrotizing soft tissue infections
        iv. Non-limb location of cellulitis, such as face, abdominal wall or perianal region
        v. Surgical site infection
        vi. Water exposure as suspected portal of entry for skin and soft tissue infection
        vii. Wound/ulcer associated with skin and soft tissue infection (e.g. diabetic foot infection)
        viii. Bursitis
2. **Classify type of infection**
    a. Purulent (likely *Staphylococcus aureus*) with or without incision and drainage
        i. Oral options: TMP/SMX, doxycycline, minocycline, clindamycin, and linezolid
        ii. If patient improved with only anti-MSSA coverage, cephalexin, cefadroxil or dicloxacillin would be options
        iii. If I&D was performed, use wound culture to assess antibiotic selection
    b. Non-purulent (likely beta-hemolytic Streptococci)
        i. Oral options: amoxicillin, cephalexin, cefadroxil, clindamycin, dicloxacillin, penicillin, TMP/SMX
        ii. In the presence of penetrating trauma or IV drug use, then OK to also cover for MSSA/MRSA

© 2026 Livorsi DJ et al. *JAMA Network Open*.

3. **Assess <u>antibiotic selection</u> at discharge, based on infection type, microbiology results, and local guidelines**
    a. *Unnecessary*: antibiotic therapy was prescribed at discharge but not needed either because the patient had already completed a full course or did not have a bacterial infection
    b. *Inappropriate*: the prescribed antibiotic at discharge was not active against the isolated or suspected pathogen
    c. *Sub-optimal*: the use of a broad-spectrum antibiotic when a narrow agent would have sufficed, based on micro results or empiric guidance (see #2) AND the patient's know allergy history.
    d. *Optimal*: none of the above definitions (a-c) are met AND antibiotics were active against the isolated or suspected pathogens
4. **Assess prescribed <u>antibiotic duration</u> at discharge, based on infection type, clinical response, microbiology results and local guidelines.**
    **Account for inpatient duration of therapy when assessing duration prescribed at discharge*

    **Total recommended antibiotic duration for skin and soft tissue infections will be 5-10 days.*

    <u>Use the following criteria when assessing antibiotic duration:</u>
    a. If antibiotic selection was deemed "unnecessary" or "inappropriate," then choose "Not applicable."
    b. If duration is +/- 1 day of the recommended duration, then classify as "optimal"
    c. If duration is ≥ 2 days of the recommended duration, then classify as "too long"
    d. If duration is < 2 days of the recommended duration, then classify as "too short"

INTRA-ABDOMINAL OR BILIARY TRACT INFECTION

1. **Apply manual exclusion criteria**
    a. Diagnostic code was wrong, and there is no infection
    b. Neutropenia (ANC < 500)
    c. Admitted from an outside hospital and antibiotic therapy prior to transfer is not known
    d. Discharged with IV antibiotics
    e. Source control not achieved, if relevant
    f. Abscess(es) with or without percutaneous drainage
    g. Secondary peritonitis due to a perforated viscus
    h. Infection at a different body site that is also being treated with antibiotics
    i. Bloodstream infection due to *Candida*, *Enterococcus* species or *Staphylococcus aureus*
2. **Classify infection based on documented signs/symptoms, laboratory data, and radiologic studies**
    a. Appendicitis: Nonperforated appendicitis or acute perforated s/p appendectomy
    b. Cholangitis: cholangitis s/p drainage procedure of biliary tract or s/p spontaneous passage of stone
    c. Cholecystitis: s/p cholecystectomy or s/p cholecystostomy tube
    d. Diverticulitis without abscess
    e. Spontaneous bacterial peritonitis
3. **Assess <u>antibiotic selection</u> at discharge, based on infection type, microbiology results, and local guidelines**
    a. *Unnecessary*: antibiotic therapy was prescribed at discharge but not needed either because the patient had already completed a full course or did not have a bacterial infection
    b. *Inappropriate*: the prescribed antibiotic regimen was not active against suspected pathogens (in the absence of cultures) or the isolated pathogen (exceptions include intentional non-coverage of *Enterococcus* species or Candida species)

© 2026 Livorsi DJ et al. *JAMA Network Open*.

c. *Sub-optimal*: not applicable to intra-abdominal and biliary infections
d. *Optimal*: none of the above definitions (a-c) are met (i.e. antibiotics were active against the isolated or suspected pathogens)

Empiric antibiotic options

- **The below list is not comprehensive. Use your clinical judgement if the prescribed agent isn't on this list.* Amoxicillin-clavulanate (especially in patients with no recent antibiotic exposure)
- Cefdinir plus metronidazole
- Ciprofloxacin or levofloxacin plus metronidazole

4. **Assess prescribed <u>antibiotic duration</u> at discharge, based on infection type, clinical response, micro results**

**Account for inpatient duration of therapy when assessing duration prescribed at discharge*

Type of infection	***Total antibiotic duration***
Appendicitis<br>Nonperforated<br>Perforated s/p appendectomy	 *7-10 days*<br>*4-7 after operation*
Cholangitis s/p biliary decompression	*4-7 days after source control*
Cholecystitis<br>Cholecystitis s/p cholecystectomy<br>Cholecystitis s/p cholecystostomy tube	 *No more than 1 day after surgery*<br>*4-7 days after source control*
Diverticulitis<br>Diverticulitis without abscess	 *7-10 days*
Secondary peritonitis from perforated viscus (now repaired)	*4-7 days*
Spontaneous bacterial peritonitis	*5-7 days (don't count prophylaxis)*

<u>Use the following criteria when assessing antibiotic duration:</u>

a. If antibiotic selection was deemed "unnecessary" or "inappropriate," then choose "Not applicable."
b. If duration is +/- 1 day of the recommended duration, then classify as "optimal"
c. If duration is ≥ 2 days of the recommended duration, then classify as "too long"
d. If duration is < 2 days of the recommended duration, then classify as "too short"

© 2026 Livorsi DJ et al. *JAMA Network Open*.

eTable 1. Timeline of study pre-implementation and implementation activities

Dates	Activities
September – December 2021	Sites were recruited to participate.
January – June 2022	Study team collected baseline data from all participating sites.
July 2022	The study team started to have a monthly collaborative learning call with all participating sites. These monthly calls continued until the trial ended in November 2023.
July-August 2022	Collaborative calls were held with participating sites to discuss the feasibility of implementing different types of strategies at hospital discharge. Each site responded to a survey about the local feasibility of different potential strategies.
September 2022	All sites reached consensus on the structure and timeline of the intervention.
November 2022	The main study team shared data with each site on their baseline antibiotic-prescribing trends.
December 2022-January 2023	Sites developed a local plan for discharge-focused audits and developed oral antibiotic stepdown guidelines, if not already implemented.
February 2023	All sites presented their plans for discharge-focused audits during a collaborative learning call.
March 2023	First site notified of their start date two months in advance
May 2023	First site was activated
November 2023	Trial ended.
November-December 2023	A post-intervention electronic survey was sent to prescribers at each site who the antibiotic stewardship team frequently interacted with as part of the discharge-focused intervention.

© 2026 Livorsi DJ et al. *JAMA Network Open.*

eTable 2. Processes used at each hospital for identifying patients who would possibly be discharged on oral antibiotics and for giving feedback to prescribers

	Participating hospital									
	1	2	3	4	5	6	7	8	9	10
How will eligible patients be identified by the stewardship team and/or clinical pharmacists who are involved in the discharge-focused audits?	By the on-floor Med Specialist pharmacist	Epic screening report and filter for intervention unit, discharge time (<2 days), and condition	Epic screening report and filter for intervention unit, discharge time (<2 days), and condition	Epic screening report and filter for intervention unit, discharge time (<2 days), and condition	Microsoft Teams chat (IDT Huddle) and use of discharge pharmacists	Automated teams chat sent to inpatient teams @ 1500 daily (M-F, and 0700 Monday mornings to identify that day's discharges)	Team pharmacist at time of patient discharge	Screening Epic report for pending discharges in patients on active antibiotics. Discharge pharmacist helped screen for patients they had not received infor-mation on from the AS team.	Epic screening report and filter for intervention unit, discharge time (<2 days), and condition	Epic screening Report and filter for intervention unit, discharge time (<2 days), and condition
Who will be involved in reviewing the EMR to identify opportunities to improve antibiotic therapy at discharge?	Med specialist pharmacist and AS Pharmacist	AS pharmacist and other Staff pharmacist	TOC pharmacists, clinical pharmacists, other ID pharmacists, and AS physicians	AS pharmacist/ and Floor clinical specialist	AS pharmacist	AS physician and/or AS pharmacist performed a focused discharge review before discharge med reconciliation is completed	Team pharmacist and perhaps ID attending on ID service at that time if rec is not accepted by the team	AS physician and AS pharmacist	Resident, AS, and Clinical pharmacists	AS and Saff pharmacists
When will discharge-focused audits be done?	Every weekday	Every weekday	Every weekday	3+ days a week on weekdays	Mon-Fri 0800-1530, preferably in the morning.	Every weekday morning	At the time of discharge planning every weekday	Three days per week, normally Mon, Wed, and Fri	Weekdays, day-shift	Every weekday
When and how will feedback from the audits be provided to the primary prescriber?	1: in-person<br><br>2: Epic chat	1: Epic chat<br><br>2: in-person	1: Epic chat<br><br>2: in-person	1: in-person during multi-disciplinary rounds<br><br>2: Epic Chat<br><br>3: telephone	1: in-person by team pharmacists<br><br>2: through EMR or by phone or Microsoft Teams chat.<br><br>In addition, AS note in EMR documented recs and alerted providers.	On the morning of the day of discharge, the provider received recs via Microsoft Teams chat and an AS note written in EMR	Feedback was provided in-person to prescribers at the time of discharge planning	1: Voalte messaging system<br><br>2: paging<br><br>3: in-person<br><br>An estimated half of recs were made in-person.	1: Texting<br><br>2: Epic chat<br>3: In-person	1: Epic chat<br><br>2: in-person

Abbreviations: AS antibiotic stewardship; EMR electronic medical record; ID Infectious Disease; IDT interdisciplinary team; TOC transition of care.

© 2026 Livorsi DJ et al. *JAMA Network Open.*

eTable 3. Fidelity to and time commitment of the intervention, based on weekly surveys completed by each site during the intervention period

	Site 1	Site 2	Site 3	Site 4	Site 5	Site 6	Site 7	Site 8	Site 9	Site 10
**Number of weeks doing the intervention**	26	24	22	20	18	16	14	12	10	8
**Number of days audits were done per week, average**	4.2	4.8	4.4	3.8	4.8	4.8	5.0	3.4	4.3	4.7
**% of weeks when at least one audit was done**	100	100	100	100	100	100	86	92	100	100
**% of weeks when at least one patient was discussed with a provider**	100	100	100	100	100	100	71	83	90	100
**Personnel effort**										
Stewardship Pharmacist effort, average per week (hrs)	0.7	1.1	2.0	2.0	2.5	0.7	0.5	3.7	1.7	3.3
Clinical Pharmacist effort, average per week (hrs)	1.0	1.4	0.6	2.2	0.0	0.0	1.2	0.5	0.5	1.3
Stewardship Physician effort, average per week (hrs)	0.0	0.0	0.6	0.0	0.0	0.9	0.5	1.3	0.0	0.0
**Discharge-focused audit-and-feedback process**										
Unique patients reviewed (total for all weeks)	113	445	916	304	335	50	54	229	195	438
Unique patients discussed (total for all weeks)	90	129	157	133	137	44	47	28	17	80
Unique patients reviewed (average per week)	4.3	18.5	41.6	15.2	18.6	3.1	3.9	19.1	19.5	54.8
Unique patients discussed (average per week)	3.5	5.4	7.1	6.7	7.6	2.8	3.4	2.3	1.7	10.0
**Types of antibiotic recommendations made**										
**Antibiotic duration recommendations**										
Number made	41	76	67	80	103	33	18	16	7	46
Number accepted[1]	33	71	57	69	87	29	10	15	7	46
**Antibiotic selection recommendations**										
Number made	61	40	58	45	91	27	20	6	5	33
Number accepted[1]	59	36	52	38	83	23	19	6	5	33
**Route of administration recommendations**										
Number made	1	32	30	49	72	0	11	0	2	36
Number accepted[1]	1	31	27	43	65	0	11	0	2	36
**Antibiotic Dose**										
Number made	5	29	17	44	89	25	19	1	0	36
Number accepted[1]	5	29	16	44	81	23	19	1	0	36
**Stop antibiotics recommendations**										
Number made	1	32	33	61	18	8	10	7	7	30
Number accepted[1]	0	30	29	53	15	5	7	4	5	30

1. Recommendations were considered to be accepted if the prescriber stated an intention to follow the stewardship recommendation at the time it was delivered.

© 2026 Livorsi DJ et al. *JAMA Network Open.*

eTable 4. Number of cases excluded and included from manual chart reviews across participating units from the 10 hospitals

Site number	Number of cases excluded[1,2]		Number of cases included	
	Baseline	Intervention	Baseline	Intervention
**1**	64	43	22	21
**2**	16	19	23	20
**3**	50	30	30	21
**4**	15	15	14	10
**5**	17	5	30	20
**6**	8	12	30	20
**7**	15	11	30	20
**8**	12	24	30	17
**9**	30	5	29	6
**10**	25	11	26	15
**Total**	252	175	264	170

1. Cases were excluded based on criteria outlined in the protocol for manual chart reviews (see pages 2-11).
2. Reasons for exclusion were complicated infection, as defined by the protocol (n=318); a second bacterial infection requiring antibiotic therapy (n=41); solid organ transplant recipient (n=22); no infection was present, i.e., the ICD-10 code was incorrect (n=20); intravenous antibiotics prescribed at hospital discharge (n=14); neutropenia (n=5); key data elements were missing from the medical record (n=3).

© 2026 Livorsi DJ et al. *JAMA Network Open.*

eTable 5. Findings from manual chart reviews of 434 patients across participating units from the 10 hospitals during the baseline and intervention periods

	Baseline period (n=264)	Intervention period (n=170)
**Optimal selection and duration, n (%)**	122 (46.2)	100 (58.8)
**Selection, n (%)**		
Optimal	204 (77.3)	143 (84.1)
Sub-optimal	21 (8.0)	12 (7.1)
Undertreatment	8 (3.0)	8 (4.7)
Unnecessary	31 (11.7)	7 (4.1)
**Duration, n (%)**		
Optimal	134 (50.8)	105 (61.8)
Too long	4 (1.5)	1 (0.6)
Too short	87 (33.0)	49 (28.8)
Not applicable	39 (14.8)	15 (8.8)
**Types of infections included, n (%)**		
Skin and soft tissue	41 (15.5)	38 (22.4)
Urinary tract, including asymptomatic bacteriuria	61 (23.1)	48 (28.2)
Intra-abdominal and/or biliary tract	38 (14.4)	16 (9.4)
Respiratory tract	124 (47.0)	68 (40.0)

© 2026 Livorsi DJ et al. *JAMA Network Open.*

eTable 6. Findings from a post-intervention electronic survey of frontline antibiotic prescribers at all ten participating sites

	Site 1	Site 2	Site 3	Site 4	Site 5	Site 6	Site 7	Site 8	Site 9	Site 10	Total
**# Responses**	2	2	5	5	5	4	9	7	0	1	40
**# Survey's Sent**	7	7	14	15	14	8	25	8	6	8	112
**Percent Response**											35.7%
**Average # of Approaches by Team, n (%)**											
**Never**	1 (50)	0	0	2 (40)	0	0	0	1 (14)	0	0	4 (10)
**<1/Week**	0	0	0	0	0	0	0	2 (29)	0	0	2 (5)
**~1/week**	0	2(100)	4 (80)	3 (60)	0	0	4 (44)	0	0	0	13 (32.5)
**2-3x/Week**	0	0	1 (20)	0	3 (60)	4(100)	3 (33)	2 (29)	0	1 (100)	14 (35)
**Nearly every day**	1 (50)	0	0	0	2 (40)	0	2 (22)	2 (29)	0	0	7 (17.5)
**The frequency that the stewardship team's suggestions were made in time for antibiotic changes to be made prior to discharge, n (%)**											
**Never**	0	0	0	1 (33)	0	0	0	1 (17)	0	0	2 (5.6)
**Seldom**	0	0	0	0	0	0	0	0	0	1 (100)	1 (2.8)
**~1/2 Time**	0	0	1 (25)	0	0	0	1 (11)	2 (33)	0	0	4 (11.1)
**Usually**	0	2(100)	0	1 (33)	1 (20)	3 (75)	3 (33)	0	0	0	10 (27.8)
**Always**	1(100)	0	3 (75)	1 (33)	4 (80)	1 (25)	5 (56)	3 (50)	0	0	19 (52.8)
**The stewardship team's suggestions helped improve decision-making about antibiotic-prescribing at discharge, n (%)**											
**Never**	0	0	0	0	0	0	0	1 (17)	0	0	1 (2.8)
**Seldom**	0	0	0	0	0	0	0	0	0	1 (100)	1 (2.8)
**~1/2 Time**	0	0	0	0	0	0	1 (11)	0	0	0	1 (2.8)
**Usually**	1(100)	0	2 (40)	2 (66)	3 (60)	2 (50)	4 (44)	3 (50)	0	0	17 (47.2)
**Always**	0	2(100)	3 (60)	1 (33)	2 (40)	2 (50)	4 (44)	2 (33)	0	0	16 (44.4)
**The stewardship team improved antibiotic prescribing at discharge through their initiative, n (%)**											
**Strongly Disagree**	0	0	0	0	0	0	0	1 (17)	0	0	1 (2.8)
**Disagree**	0	0	0	0	0	0	0	0	0	0	0 (0)
**Neutral**	0	0	0	0	0	0	0	0	0	1 (100)	1 (2.8)
**Agree**	0	1 (50)	1 (20)	3(100)	3 (60)	2 (50)	4 (44)	2 (33)	0	0	16 (44.4)
**Strongly Agree**	1(100)	1 (50)	4 (80)	0	2 (40)	2 (50)	5 (56)	3 (50)	0	0	18 (50)
**The stewardship team should continue their initiative to review and provide feedback on antibiotic-prescribing at discharge, n (%)**											
**Strongly Disagree**	0	0	0	0	0	0	0	1 (17)	0	0	1 (2.9)
**Disagree**	0	0	0	0	0	0	0	0	0	0	0 (0)
**Neutral**	0	0	0	0	0	1 (25)	1 (11)	0	0	1 (100)	3 (8.6)
**Agree**	0	0	1 (20)	1 (50)	3 (60)	0	4 (44)	1 (17)	0	0	10 (28.6)
**Strongly Agree**	1(100)	2(100)	4 (80)	1 (50)	2 (40)	3 (75)	4 (44)	4 (66)	0	0	21 (60)

© 2026 Livorsi DJ et al. *JAMA Network Open.*

© 2026 Livorsi DJ et al. *JAMA Network Open*.
