# Supplementary material for: Prospective Audit and Feedback by Antibiotic Stewardship Teams to Reduce Antibiotic Overuse at Hospital Discharge: A Stepped-Wedge Cluster-Randomized Clinical Trial
Source: JAMA Netw Open. 2026 Jan 9;9(1):e2549655. doi: 10.1001/jamanetworkopen.2025.49655 (PMC12789953; doi:10.1001/jamanetworkopen.2025.49655)
Supplement: Supplement 3. — Data Sharing Statement [file jamanetwopen-e2549655-s003.pdf]

Data Sharing Statement

Livorsi. Prospective Audit and Feedback by Antibiotic Stewardship Teams to Reduce Antibiotic Overuse at Hospital Discharge. *JAMA Netw Open*. Published January 09, 2026.
doi:10.1001/jamanetworkopen.2025.49655

Data
Additional Information: clinicaltrials.gov #NCT05471726
Data available: No

Additional Information
Explanation for why data not available: Three of the participating hospitals were from the VA, and the VA does not allow us to share patient data that was collected from their Corporate Data Warehouse.
